# Varicose: a MAGUK required for the maturation and function of *Drosophila *septate junctions

**DOI:** 10.1186/1471-213X-8-99

**Published:** 2008-10-10

**Authors:** Katherine E Moyer, J Roger Jacobs

**Affiliations:** 1 Department of Biology, McMaster University, 1280 Main St. W. Hamilton, Ontario, Canada, L8S 4K1

## Abstract

**Background:**

Scaffolding proteins belonging to the membrane associated guanylate kinase (MAGUK) superfamily function as adapters linking cytoplasmic and cell surface proteins to the cytoskeleton to regulate cell-cell adhesion, cell-cell communication and signal transduction. We characterize here a *Drosophila *MAGUK member, Varicose (Vari), the homologue of vertebrate scaffolding protein PALS2.

**Results:**

Varicose localizes to pleated septate junctions (pSJs) of all embryonic, ectodermally-derived epithelia and peripheral glia. In *vari *mutants, essential SJ proteins NeurexinIV and FasciclinIII are mislocalized basally and epithelia develop a leaky paracellular seal. In addition, *vari *mutants display irregular tracheal tube diameters and have reduced lumenal protein accumulation, suggesting involvement in tracheal morphogenesis. We found that Vari is distributed in the cytoplasm of the optic lobe neuroepithelium, as well as in a subset of neuroblasts and differentiated neurons of the nervous system. We reduced *vari *function during the development of adult epithelia with a partial rescue, RNA interference and generation of genetically mosaic tissue. All three approaches demonstrate that *vari *is required for the patterning and morphogenesis of adult epithelial hairs and bristles.

**Conclusion:**

Varicose is involved in scaffold assembly at the SJ and has a role in patterning and morphogenesis of adult epithelia.

## Background

The assembly of cellular junctions is pivotal for metazoans to maintain a homeostatic environment. Through these junctions, cells are able to communicate, synchronize function, and regulate the paracellular flow of molecules [1-3]. Epithelial cells are polarized along an apico-basal axis where the apical surface faces the exterior or lumen and the basal surface communicates with the extracellular matrix [4]. In vertebrates, several epithelial intercellular junctions exist, the two most widely studied being tight junctions (TJs) and adherens junctions (AJs) [5,6]. Invertebrate species, although lacking tight junctions possess the functionally analogous septate junction (SJ) [7]. Despite a difference in lateral membrane location, TJs (apical to AJs) and SJs (basal to AJs) both form an intercellular barrier to regulate the transepithelial diffusion of solutes [8].

Ultrastructure and freeze-fracture analysis of cell junctions reveal that SJs maintain a fixed distance between epithelial cells through ladder-like septae that spiral on the outside of the cell and fill the intermembrane space [7]. These encircling septae extend the travel distance for molecules to transverse the paracellular path, thereby regulating the flow of material [9]. TJs however, appear as a series of contact points, or 'kissing sites'. Freeze-fracture analysis reveals that TJs consist of interconnecting mesh-works of fibrils forming a band-like structure around the cell. In spite of a diverged morphology, globular and transmembrane proteins are suspected to form the bridge between cells filling the intermembrane space of TJs and SJs, respectively [8,10].

Two types of SJs have been observed in *Drosophila*, smooth (sSJs) and pleated (pSJs). Smooth SJs, which lack ladder-like septa are found in tissues such as the midgut and malpighian tubules. Pleated SJs are found in all ectodermally-derived epithelial tissues such as trachea, salivary glands, hindgut and epidermis as well as in glial cells [8]. Glial SJs function to link ensheathing glial cells around peripheral nerves, forming the blood-brain barrier [11,12]. Recently, SJ were indentified in the apical and basal regions between accessory cells, the cone cells and pigment cells of adult ommatidia [13]. SJs and TJs, both in epithelia and neurons, share core components suggesting that barrier function is a conserved mechanism between vertebrate and invertebrate species [2].

Within the last decade, key molecular elements of pSJs have been identified and shown to be involved in processes such as establishing and maintaining cell polarity, cell adhesion and cell-cell interactions [8]. Coracle (Cora), NeurexinIV (NrxIV), Neuroglian (Nrg), Na^+^/K^+ ^ATPase (ATPα), and Discs Large (Dlg) have all been identified as SJ constituents [14-17]. *Drosophila *Coracle, a member of the Protein 4.1 family, interacts with transmembrane proteins forming a link to the cytoplasmic surface of the plasma membrane [18]. Cora localizes to epithelial pSJs, where it is required for SJ organization but is absent from the CNS and its derivatives [16,19]. Interactions between Cora and transmembrane protein NrxIV are necessary in order to maintain their proper SJ localization [19]. NrxIV, a member of the neurexin gene family, localizes to all pSJs of ectodermally-derived epithelia and the CNS [15]. In both *cora *and *nrxIV *mutants, intermembrane septae are absent, resulting in a leaky paracellular seal. While Cora and NrxIV have been found to interact with ATPα at the SJ, they have also been shown to form a complex independently with Nrg. Nrg is an integral membrane glycoprotein that localizes to the lateral membrane of epithelial cells and to the surface of glial cells, regulating the adhesion between neurons and glial cells [20,21]. Mutations in either ATPα or Nrg results in mislocalization of Cora and NrxIV and disrupts SJ structure and function. This suggests that interdependent protein complexes function to assemble the protein scaffold regulating paracellular movement. However, among these mutants, epithelial integrity, apico-basal polarization and the localization of SJ protein Dlg are unaffected [18].

**M**embrane **A**ssociated **GU**anylate **K**inases, MAGUKs, are a class of scaffolding proteins that tether adhesion molecules at sites of cell-cell contact, such as septate and tight junctions [22]. MAGUK proteins contain a core domain structure consisting of 1–3 PDZ domains (named after 3 founding MAGUK proteins **P**SD-95, **D**lg and **Z**O-1), a src homology 3 (SH3) domain and a guanylate kinase domain (GUK) [23]. In addition, some MAGUK members encode an N-terminal L27 domain (named after interacting proteins Lin-2 and Lin-7) which functions in protein-protein interactions [24]. This multi-domain composition allows MAGUKs to function as the backbone onto which protein complexes can assemble [25]. These complexes then bring together functionally dissimilar proteins to link transmembrane proteins with the cytoskeleton [26].

The PALS (**P**roteins **A**ssociated with **L**in-7) subfamily of MAGUK proteins, PALS1 and PALS2, anchor scaffolding complexes at junctional regions [22,27]. *Drosophila *Stardust (Sdt) and its homologue, vertebrate PALS1, function as adapter proteins linking two scaffolding complexes to establish epithelial polarity [28,29]. The functional significance of PALS2 remains unclear. Vertebrate studies have shown that PALS2 interacts with the C-terminus of Nectin-like molecule 2 (Necl-2) through its PDZ domain at spot-like adhesion sites along the lateral plasma membrane. Furthermore, Necl-2 binds DAL1, a Protein 4.1 family member, implicating PALS2 in membrane organization and scaffold assembly [30]. The evolutionary conservation of junctional proteins prompted our search for the invertebrate homologue of PALS2 to elucidate its molecular and genetic function. Our search identified *Drosophila *CG9326, also reported recently as the gene interrupted in the v*aricose *(*vari*) mutation [31,32]. Our previously reported s*enz'aria *(*szar*) alleles [33] are thus renamed as alleles of *vari*.

We present here our findings of *Drosophila *CG9326, Varicose (Vari), a homologue of vertebrate PALS2. Previous independent studies identify a function for Vari in the function of epithelial SJs [32,34]. Our studies show that Vari localizes to the SJ in all embryonic ectodermally-derived epithelial tissues as well as glial cells of the PNS. Mutations in *vari *result in mislocalized SJ markers such as NrxIV and compromise the seal of the transepithelial barrier. We have identified Vari expression in larval optic lobe neuroblasts and in the adult nervous system. Furthermore, Varicose is required for the patterning of adult epithelial structures. Adult wing hair extension and orientation, patterning of thoracic bristles, and the patterning of inter-ommatidial bristles are disrupted when *vari *function is reduced or removed. This study identifies both conserved and novel functions for MAGUK proteins in *Drosophila *development.

## Results

### Varicose, a homologue of vertebrate PALS2/VAM-1

**Figure 1:**
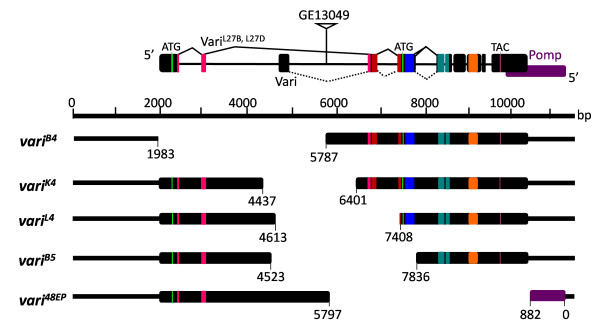
**Varicose alleles created by imprecise excision**. Imprecise excision of P-element insertion line GE13049 generated an allelic series of *vari*. Five alleles were produced, *vari*^*B*4^, *vari*^*K*4^, *vari*^*L*4^, *vari*^*B*5 ^and *vari*^48*EP*^. Excision *vari*^48*EP *^generated a protein null allele. The 3'UTR of *vari *overlaps with the 3'UTR of the adjacent *CG9324/pomp*. The *vari*^48*EP *^excision removes the 3'UTR's of both genes, and three terminal amino acids of CG9324. Start/stop codons and protein domains are colour coded as follows: start sites (green), L27N and L27C domains (pink and red. see[34]), PDZ domain (blue), SH3 domain (teal), GUK domain (orange), stop codon (purple).

Our search for the invertebrate homologue of mammalian PALS2 identified *Drosophila *CG9326 as a candidate, sharing 39% amino acid identity to PALS2/VAM-1. Like its vertebrate counterparts, CG9326 encodes a MAGUK protein possessing PDZ, SH3 and GUK domains. The genomic sequence of CG9326 is 8381 bp in length and is composed of 10 introns and 11 exons [35]. Genome annotations predict that CG9326 may generate three transcripts, denoted B, C and D, which would encode three proteins, Vari^L27B^, Vari^L27D ^and Vari (Fig. 1). Transcripts B and D, but not C give rise to products encoding two N-terminal L27 domains, as previously described [34]. L27 domains interact heterophilically to link scaffolding proteins, for example Lin-2 (CASK) and Lin-7 (Veli) [36]. CG9326-B differs from CG9326-D by a 21 amino acid insertion, as vertebrate PALS2β differs from PALSα by a 14 amino acid insertion [27].

### Varicose localizes to embryonic epithelial tissues

The embryonic expression of Vari has been previously described [32]. We detected *varicose *transcripts from early stage 10 of embryogenesis until hatching. Protein expression was first detected during stage 13 of embryogenesis by immunolabeling with anti-Vari, and until late stage 17.

**Figure 2:**
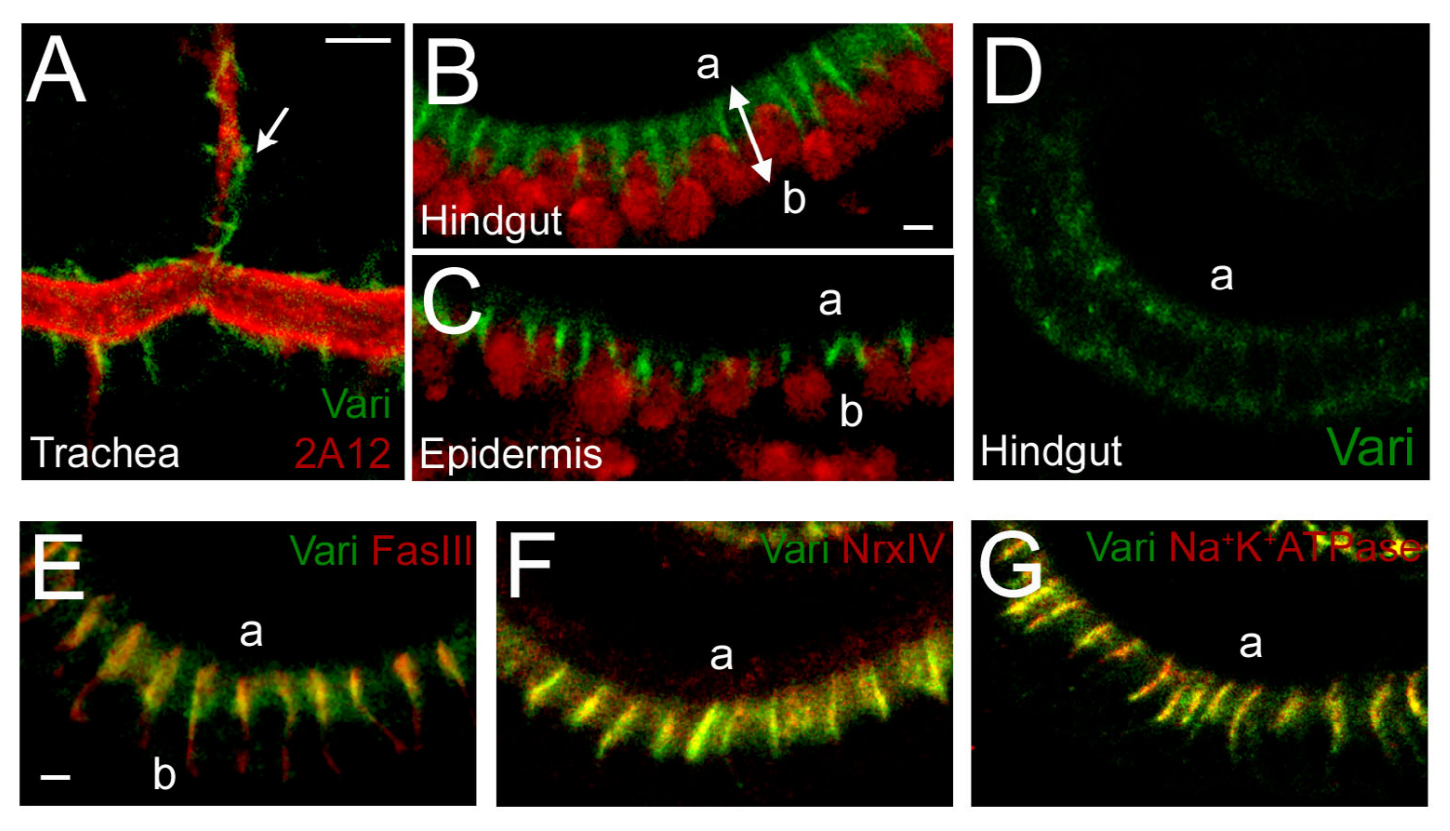
**Varicose is detected at the septate junction in ectodermally-derived tissues**. (A) WT embryos stained with Vari (green) and tracheal antibody, 2A12 (red). Vari is restricted to epithelial cells and is excluded from tracheal lumen (arrow). (B-C) WT embryonic hindgut (B) and epidermis (C) labeled with Vari (green) and nuclear stain, propidium iodide (red). Vari is limited to the basolateral region of the plasma membrane in both tissues. (D) Vari was not detected at the SJ in the null allele, *vari*^48*EP*^. (E-G) Confocal microscopy was used to visualize the hindgut of WT embryos, labeled with Vari (green) and SJ markers (red). Vari is restricted to SJs, shown by co-localization with SJ markers FasIII (E), NrxIV (F), and Na^+^K^+^ATPase (G). Apical membrane faces are indicated (a) and basal (b). All embryos are stage 15. WT, wildtype. Calibration: 5 μm, A; 2 μm, B-G.

In concurrence with the previous study, we found Varicose expression to be restricted to epithelial tissues such as the epidermis, trachea, proventriculus, salivary gland, and hindgut (see Additional file 1, panel A), [32]. Subcellular labeling was restricted to the lateral membrane in the trachea (arrow, Fig. 2A), hindgut (Fig. 2B), and epidermis (Fig. 2C). We did not detect expression in non-ectodermal epithelia, such as the amnioserosa or malphigian tubules, or in any epithelial tissues in our null allele, *vari*^48*EP *^(Fig. 2D, see Additional file 2, panel A).

### Varicose localizes to the septate junction during embryogenesis but not in imaginal discs

In *Drosophila*, MAGUKs typically function as scaffolding proteins upon which multiprotein complexes form to regulate cell polarization and adhesion (reviewed in Funke et al., [22]). The restricted membrane localization of Vari suggested to us that it may act similarly. We compared Varicose expression with various lateral membrane markers in the hindgut of stage 15 embryos (Fig. 2E–G, see Additional file 1, panels B-G). Vari localizes adjacent to, yet fails to co-localize with, the sub-apical marker Crumbs [37] and the adherens junction marker Phosphotyrosine [38]. Co-localization of Varicose and plasma membrane marker α-Spectrin [21] is seen in the apical region of the lateral membrane but Vari is not seen in the basal region, indicating Vari localizes to the apicolateral membrane, a region corresponding to the SJ. Septate junctions are characterized by the localization of proteins such as Discs-large, Coracle and NeurexinIV [14-16]. Double-labeling experiments with Varicose and SJ markers FasIII (Fig. 2E), NrxIV (Fig. 2F) and Na^+^K^+ ^ATPase (Fig. 2G) reveal a complete overlap of expression, suggesting that Varicose is localized solely in the septate junction. Co-localization of Vari and Dlg is also seen in the trachea, salivary gland and proventriculus (data not shown). In concurrence with a previous study, our evidence indicates that Varicose expression is restricted to the SJ region in all embryonic ectodermally-derived epithelial tissues [32].

If Vari is required by epithelial SJ, then it should be expressed in imaginal epithelia. Although Bachmann et al.,[34], report immunolabeling of eye and wing discs, our antibody did not reveal a labeling pattern different from controls (see Additional file 3). Phenotype data explored below suggests that Vari does function in imaginal epithelia.

### MAGUK function in the nervous system

**Figure 3:**
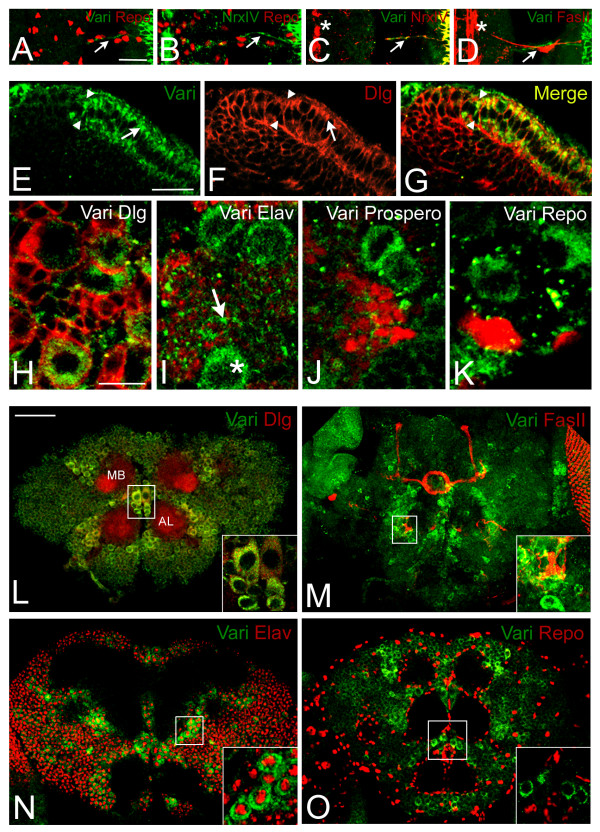
**Varicose nervous system expression is novel for a MAGUK**. (A-D) Stage 16–17 wildtype embryos were stained with Vari and glial cell marker, Repo. Vari localizes with Repo in peripheral glial cells (arrow, A) in a similar pattern to NrxIV, a known pSJ marker of PNS glia (arrow, B). Embryos labeled with Vari and NrxIV show co-localization of these proteins in peripheral glia (arrow, C) but not in midline glia (asterisk, C). No Vari labeling was found in the CNS (at left of A, asterisk, C-D). NrxIV and Vari label at right is in the ectoderm. Vari does not label developing synapses identified using Fasciclin II antibody (arrow, D). Ventral views from a single confocal section. Anterior at top; ventral midline at left. Brains from late 3^rd ^instar larva were labeled with Vari and Dlg (E-G). (E) Vari expression is concentrated in the apical region (arrow) of neuroepithelial (NE) cells (right of arrowheads) and excluded from neuroblasts (NBs) (left of arrowheads). (F) Dlg is concentrated at the SJ of NE cells (arrow) and distributed cortically in NBs. Co-localization of Dlg and Vari is limited along the lateral membrane of the NE (G). (H-K) Vari (green) is detected throughout the cytoplasm in a subset of central brain neuroblasts. Vari fails to co-localize with Dlg at the cortex (red, H) and appears to have low levels of expression in Elav expressing post-mitotic neurons (arrow, Elav, I). Vari is excluded from ganglion mother cells that label for Prospero (red, J), and glial cells that label for Repo (red, K) (NBs denoted by asterisk). (L-O) Pupal brains stained with Vari (green) and Dlg (L), FasII (M), Elav (N) or Repo (O) (red). (L, N, O) Single cross sections visualized by confocal microscopy. Vari and Dlg co-localize in cell bodies (inset, L) but fail to co-localize in the antennal lobes (AL) and mushroom bodies (MB). (M) A projection of sections visualized by confocal microscopy. Vari is excluded from the MB and axon tracts labeled by FasII. Vari expression appears to concentrate in areas surrounding the axon tracts (inset, M). Vari localizes to cells expressing Elav (inset, N) but is not expressed in glial cells labeled with Repo (inset, O). Calibration: 20 μm, A-D and E-G; 10 μm, H-K; 50 μm, L-O.

If Varicose is localized to pSJs, then we would predict that SJs found in glial cells of the peripheral nervous system (PNS) would express Vari. Varicose is localized to cell junctions in Repo positive glial cells [39] in the embryo (arrow, Fig. 3A) and in the optic stalk of the adult (data not shown). To determine if Varicose localization was restricted to the SJ, we double-labeled embryos with Vari and NrxIV, a known SJ marker of glial cells (arrow, Fig. 3B). We observed co-localization of Vari and NrxIV (arrow, Fig. 3C) in peripheral nerves but unlike NrxIV, Vari was not detected in midline glia (asterisk, Fig. 3C). We did not observe Vari expression in the developing synapses (arrow, Fig. 3D) nor in the CNS (asterisk, Fig. 3D). Thus, in the embryo, Varicose expression is restricted to pSJs.

In larvae, Varicose expression was observed in the neuroepithelium of the developing optic lobe. The optic lobe consists of two populations of cells, symmetrically dividing lateral neuroepithelial (NE) cells and asymmetrically dividing medial neuroblasts. NE cells possess similar properties as embryonic epithelial cells and express junctional markers at similar locations [40]. To determine if Varicose localized to SJs in postembryonic epithelia, we labeled third instar larvae brains with Vari and Dlg. Dlg localizes to the SJ in NE cells (arrow, Fig. 3F) and to the cortex in neuroblasts (left of arrowheads, Fig. 3E–G). In contrast to what we observed in embryonic epithelia, Varicose has limited co-localization with Dlg (Fig. 3G). Varicose expression is found in the apical cytoplasm of NE cells (arrow, Fig. 3E) but is not found in neuroblasts (left of arrowheads, Fig. 3E). We were unable to detect neuroepithelial labeling with pre-immune sera suggesting the observed pattern is due to Varicose expression (see Additional file 2, panels B-D). If this expression pattern is true for other SJ markers, we would expect to see NrxIV at the SJ of NE cells. We did not observe any NrxIV expression in NE cells or in neuroblasts; we however did detect expression in neuroblast progeny (not shown). Expression of Varicose restricted to the neuroepithelium of the optic lobe suggests a role in the symmetrically dividing cell pool. Moreover, we have identified a MAGUK member that does not always associate with the plasma membrane, suggesting a novel role for this protein in the neuroepithelium.

Central neuroblasts found in third instar larvae brains also express Varicose. We performed various double-labeling experiments using several neuroblast markers. Varicose did not co-localize with Dlg, a cortex marker (Fig. 3H), Prospero, a ganglion mother cell marker (Fig. 3J), or Repo, a glial cell marker (Fig. 3K) [39,41,42]. Weak Varicose expression was observed in differentiated neurons labeled with Elav (Fig. 3I) [43]. We concluded that Varicose expression at this stage remains in the cytoplasm of neuroblasts.

Identifying Varicose expression in neuroblasts prompted us to characterize Varicose expression in the adult nervous system. We immunolabeled pupal brains 50 hours after enclosion with Vari and either Dlg (Fig. 3L), FasII (Fig. 3M), Elav (Fig. 3N) or Repo (Fig. 3O). We did not observe Varicose expression in the mushroom bodies or in the antennal lobes. Varicose however, co-localized with Dlg in the cell bodies of neurons surrounding these neuropile regions (yellow; Fig. 3L). We deduced that these cell bodies belong to differentiated neurons as opposed to glial cells because Vari is localized with Elav (Fig. 3N) and not Repo labeled cells (Fig. 3O). Therefore, Varicose localizes to a subset of differentiated neurons surrounding neuropile regions of the adult nervous system.

### Loss-of-function varicose mutants are embryonic lethal

We have created a loss-of-function allelic series, *vari*^48*EP*^, *vari*^*K*4^, *vari*^*L*4^, *vari*^*B*4^, and *vari*^*B*5^, to determine whether Vari plays a role in septate junction assembly. The embryonic lethal P-element insertion line GE13049 (GenExel, Inc), contains an EP insertion 3507 bp downstream of the translation start site of Vari^*L*27*B *^and Vari^*L*27*D*^, and 1731 bp upstream of the translation start site of Vari (Fig. 1). Using standard procedures, we mobilized GE13049 and generated 5 mutant alleles by imprecise excision. Here we present allele *vari*^48*EP*^, an excision allele which removed 4717 bp of genomic sequence (20792456...20797173) leaving behind 416 bp of the P-element. We consider this allele is a true null, as no protein is been detected, and the excised sequence has removed all of the reading frame, except the predicted L27N domain [34]. Our other alleles, *vari*^*K*4 ^and *vari*^*L*4 ^are clean excisions that removed 1964 bp and 2795 bp of genomic sequence, respectively. In addition, *vari*^*B*4 ^removed 3806 bp leaving behind 27 bp of P-element sequence, while *vari*^*B*5 ^removed 3313 bp of genomic sequence leaving 216 bp of P-element sequence behind. All *vari *alleles are late embryonic lethal, although *vari*^*K*4 ^has escapers that die during the second instar. All alleles fail to complement the lethality of GE13049 or the *Df(2L)Exel*^7079 ^deficiency. The embryonic phenotype of GE13049 is indistinguishable from mutant *vari*^48*EP *^or trans-heterozygotes. *Df(2L)Exel*^7079 ^is a molecularly characterized deficiency deleting chromosomal region 38E6-38F3. The 3' UTR of both *varicose *and *CG9324/pomp*, a 20S maturase, overlap [44]. Our *vari*^48*EP *^excision removed the 3' UTR of *pomp *as well as 3 carboxy-terminal amino acids. This *vari *allele complements a lethal allele, *pomp*^*EY*06518^, indicating that our lethal phenotype is a result of disruption of *vari*. In addition, our sequenced revertant, *vari*^34*P*^, has a wildtype phenotype.

### Septate junction assembly requires varicose

**Figure 4:**
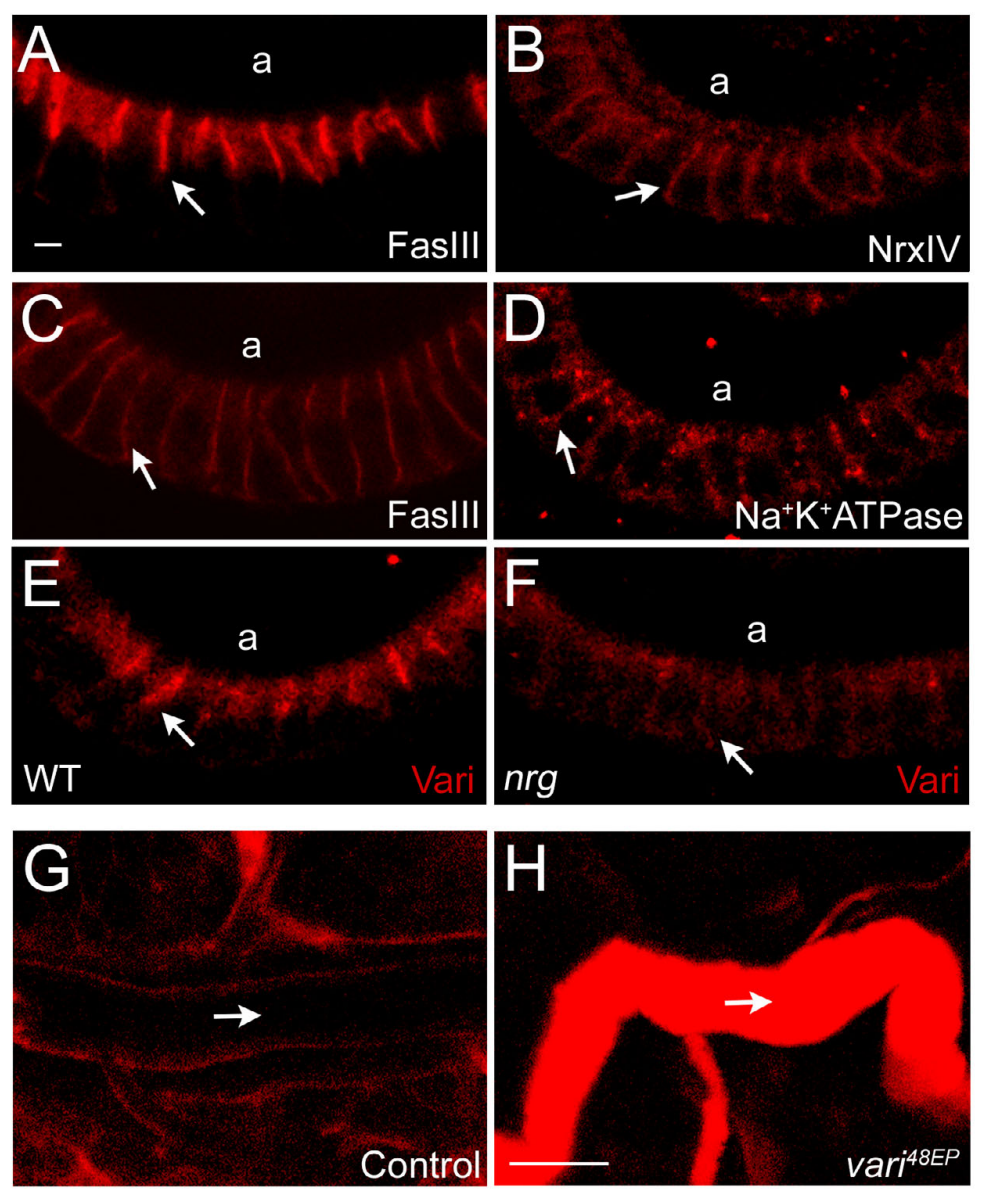
**Distribution of septate junction markers and the seal of the transepithelial barrier are compromised in *vari *mutants**. (A-D) Distribution of SJ markers in *vari *mutants. (A) Balancer LacZ control. (B, C) *vari*^*GE*13049^. (D) *vari*^48*EP*^. Homozygous embryos selected by absence of balancer lacZ and visualized by confocal microscopy. FasIII (A), NrxIV, and Na^+^K^+^ATPase (q.v. Fig. 2F, G) localize to hindgut epithelial SJs in lacZ control embryos and are excluded basally (arrow, A). In *vari *mutants, NrxIV, FasIII, and Na^+^K^+^ATPase are mislocalized basally along the lateral plasma membrane (arrows B, C, and D, respectively). (E, F) We assessed the distribution of Vari in the SJ mutant, *nrg*. In control embryos, Vari localizes to the SJ of hindgut epithelial cells (arrow, E), however accumulation of Vari is greatly reduced and mislocalized basally in *nrg *mutants (arrow, F). All embryos are stage 15. (G, H) The integrity of the transepithelial barrier in *vari*^48*EP *^mutants was determined by permeability assay as described by Lamb et al. (1998). Following injection of rhodamine-conjugated dextran, dye was detected within the tracheal lumen of mutants (arrow, H) but excluded from the lumen in controls (arrow, G), indicating disruption in barrier function. Dye remained undetectable in the lumen of controls 90 minutes post-injection. a, apical. Calibration: 2 μm, A-F; 10 μm, G-H.

Septate junction assembly begins during stage 14 of embryogenesis [7,8], a time that corresponds to the onset of Varicose expression. To determine if Varicose is required for the assembly of septate junctions, we assessed the subcellular localization of SJ markers in *vari*^48*EP *^mutants (n = 50). Proteins normally enriched at the septate junction, such as NrxIV, FasIII and Na^+^K^+^ATPase are mislocalized basally in the absence of Varicose function (arrows, NrxIV, Fig. 4B; FasIII, Fig. 4C; Na+K+ATPase, Fig. 4D). These data are consistent with the findings of Wu et al.,[32]. However, localization of SJ marker Dlg was unaffected (not shown). Discs-large is a tumor suppressor that acts to regulate cell polarization, proliferation and adhesion [14]. These data suggest that Varicose may play a role in the assembly of septate junctions but is not required for the establishment of cell polarity.

As previously mentioned, proper localization of SJ components is interdependent. We assessed the localization of Vari in several SJ mutants including *dlg*^*Xl*-2^, *nrg*^14^, *nrx*^4304^, and *cora*^*K*08713^. Unexpectedly, Vari was properly localized in all SJ mutants examined, except *nrg*^14^. Varicose expression is severely reduced and mislocalized basally along the lateral membrane (Fig. 4E, 4F). While *nrg*^14 ^mutants display reduced or absent transverse septa, the spacing between epithelial plasma membranes is maintained [17].

Septate junctions are the structural basis of the paracellular barrier in insect epithelia [45]. To determine whether the transepithelial barrier was compromised in *vari *mutants we performed dye exclusion assays, as described by Lamb et al., [18]. Rhodamine-conjugated dextran was injected into late stage wildtype embryos and dye was excluded from the lumens of the salivary glands and trachea beyond 90 minutes (Fig. 4G). In contrast, within 30 minutes of injection, dye could be detected in the tracheal lumen of *vari*^48*EP *^mutants (Fig. 4H).

**Figure 5:**
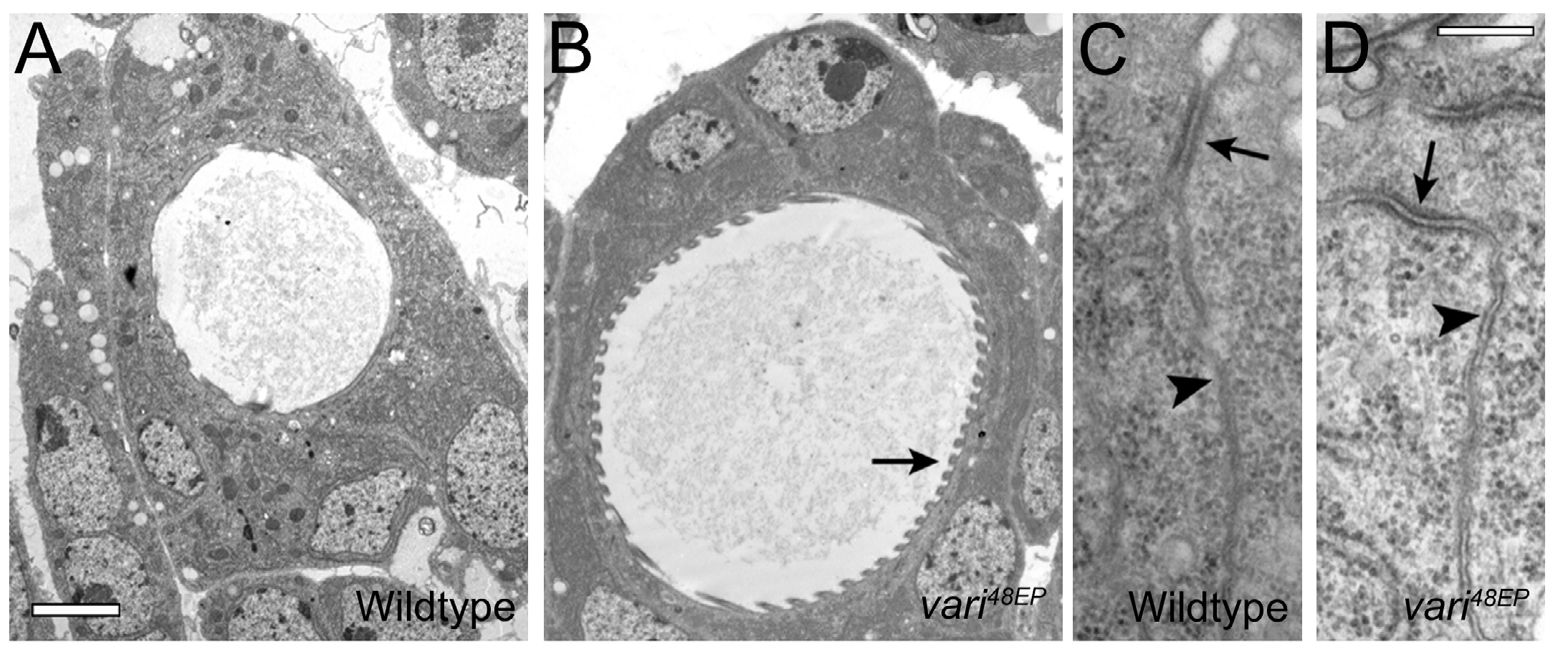
**Examination of cellular ultrastructure in *vari*^48*EP *^mutants**. (A-D) We have compared the ultrastructure of the cells of stage 16 trachea and hindgut between wildtype (A, C) and *vari*^48*EP *^mutants (B, D). The lumen of *vari *trachea could be significantly dilated relative to wildtype, but the structure of lumenal cuticle and tanideal ridges were not affected (arrow B). We also examined the ultrastructure of epithelial cell junctions from semi-serial sections of the hindgut. Relative to wildtype (C), apposed lateral membranes (arrows) and septate junctions (arrowheads) were similar in *vari *mutants (D). Calibration: 2 μm A, B and 200 nm C and D.

In order to gain insight into the structural underpinnings of barrier establishment, we examined the ultrastructure of cell junctions in *vari*^48*EP *^mutants through semi-serial electron microscopy. Structurally, the lateral membranes, in particular, the septate junction was similar in wildtype (Fig. 5C) and *vari*^48*EP *^mutants (Fig. 5D). We did not observe pleated sheets characteristic of SJs in either group. Ladder-like septa found in pleated sheets typical of mature SJs [7] do not appear in wildtype until stage 17, and at this stage, signs of necrosis indicate that *vari*^48*EP *^homozygotes are dying. This is in contrast to Bachmann et al., [34], where *vari*^03953*b *^and *vari*^*MD*109 ^embryos are not necrotic at this stage. These alleles do not eliminate the long form of Vari (transcript B), and may represent a hypomorphic phenotype. Taken together, these observations support a requirement for *varicose *in septae formation, and establishing the seal of the transepithelial barrier.

### Loss of *vari *results in dilated tracheal branches and reduced lumenal staining

**Figure 6:**
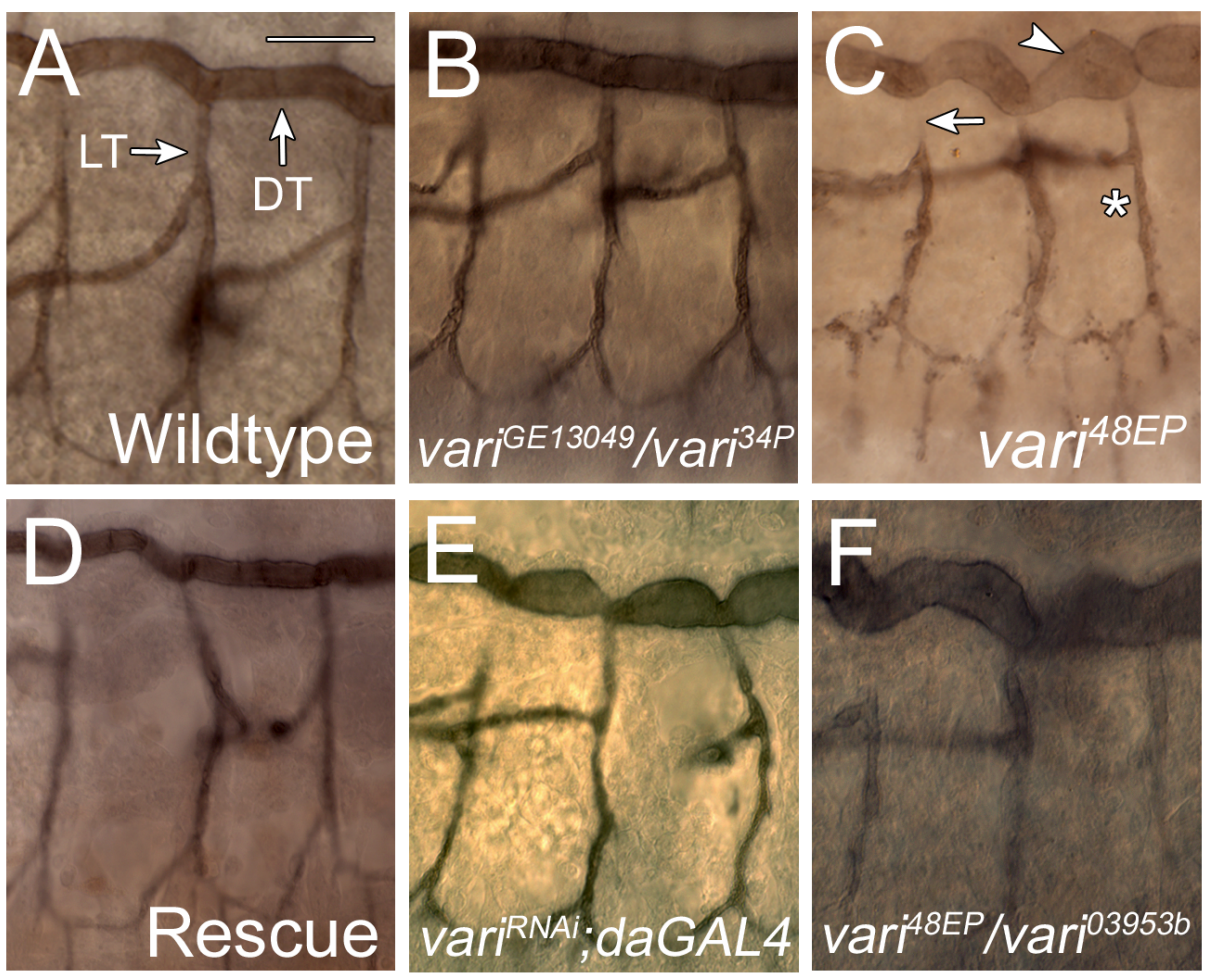
**Varicose is required for tracheal development**. (A-F) The tracheal lumen of early stage 16 *vari *mutant embryos were labeled with MAb2A12. In both WT (A) and in embryos heterozygous for the original EP insert and a precise excision of that insert (B), the diameter of the Dorsal Trunk (DT) is uniform, and the Lateral Trunk (LT) is continuous with the DT. All *vari *mutants (C and heteroallelic F), as well as *daughterless-GAL4 *mediated RNAi knockdown of *vari *(E) share similar tracheal defects. Mutants exhibit large dilations along the DT (arrowhead, C) and LT (*, C). Some LT branches appear disconnected from the DT (arrow, C). Lumenal staining is reduced in all *vari *alleles in comparison to WT and control. Tracheal phenotypes of null *vari*^48*EP *^embryos are restored upon ubiquitous expression of Vari by *daughterlessGAL4 *(D). Lateral view: anterior to the left, dorsal is up. WT, wildtype. Calibration: 20 μm, A-F.

Previous reports suggest that a disruption of septate junction assembly also reduces the efficiency of apical secretion into the tracheal lumen. This generates abnormal fibrillar structures in the lumen and distorted and tortuous tracheal trunks [46,47]. If Varicose is required for septate junction assembly, then tracheal development should also require Vari. To address this possibility, we labeled the tracheal lumen of all *vari *mutant alleles with the lumenal marker MAb2A12 (n = 200; Fig. 6). Tracheal abnormalities were found in all homozygotes of these alleles and all hetero-allelic combinations. However, tracheal morphology reverted to wildtype in *Drosophila *carrying a precise excision of *vari*^*GE*13049^, *vari*^34*P*^, and in transheterozygotes, *vari*^*GE*13049^*/vari*^34*P *^(Fig. 6B), indicating that the genetic background of the original insert did not contribute to the phenotype. Overexpression of Vari using *breathlessGAL4 *fails to rescue the tracheal phenotype (data not shown). However, ubiquitous expression using *daughterlessGAL4 *rescues the tracheal phenotype of *vari*^48*EP *^null embryos (Fig. 6D) in addition to rescuing lethality. Furthermore, the mutant tracheal phenotype was evident in transheterozygotes from different genetic backgrounds (Fig. 6F, see Additional file 4, panel H). Tracheal branches exhibit a balloon-like appearance, representing large dilations. However, tube-length was not dramatically affected. In addition, lumenal stain was reduced in all *vari *mutants (Fig. 6C, 6F, see Additional file 4, panels B-G) when compared to the controls (Fig. 6A, 6B). This is consistent with earlier findings of reduced levels of Vermiform and no Serpentine labeling in *vari *mutants [32].

To further understand the tracheal dilations, we examined tracheal cell ultrastructure using electron microscopy. Although *vari *mutants have abnormally large lumen diameters (arrow, Fig. 5B) compared to controls (Fig. 5A), the overall cell morphology is similar to wildtype (Fig. 5A and 5C). In addition, cuticle secretion was similar in both mutants and wildtype controls. Proper cuticle secretion, taenidial folds and normal cell morphology in *vari *mutants suggest that its role may be independent of apical secretion.

### Varicose acts in adult morphogenesis

A role for *vari *in the eye and wing was suggested by a partial rescue with the *vari *transgene, tissue specific protein knockdown with an inverted repeat (IR) transgene [48], and by generation of tissue mosaic for *vari *function with the FLP/FRT technique [49]. Ubiquitous expression of full-length Vari using *daughterless*GAL4 rescues lethality of *vari*^48*EP *^null embryos. Viability of the rescued animals ranges from late pupation (80%; n = 73) to viable adults (20%). Viable adults are unstable and unable to walk, and have a life span averaging 3 days. The ability to rescue *vari *mutants with a transgene lacking the L27 domain suggests that this domain is not essential for development, consistent with the finding of Bachmann et al., [34].

**Figure 7:**
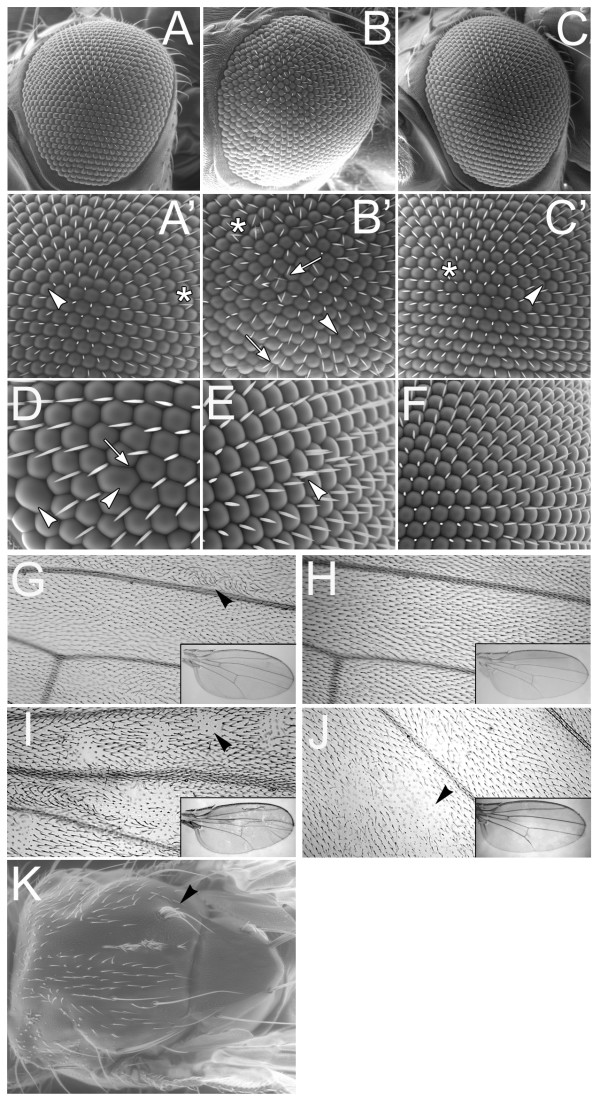
**Ommatidial patterning and wing hair alignment are abnormal in *vari *mutants**. (A-C) Ubiquitous overexpression of Vari using *daughterlessGAL4 *in heterozygous *vari*^48*EP *^(A), homozygous *vari*^48*EP *^(B) and WT backgrounds (C). (A'-C') Magnified images of A-C. Overexpression of Vari in a WT background results in mild phenotypes such as missing interommatidial bristles (IOBs) (arrowheads) or irregularly patterned bristles (below the asterisk). Overexpression of Vari in a null mutant background results in missing ommatidia and extra IOBs (arrows, B'). (D-F) When the FLP/FRT mosaic approach is used to generate patches of *vari*^48*EP *^null tissue in the developing eye disc, mutant cells are not seen in the adult eye, although fused ommatidia (arrowhead, D), missing bristles (arrow, D) and duplicated bristles (arrowhead, E) are seen. Heat-shocked siblings, in which all cells are WT for *vari*, are normal (F). (G-J) No phenotypic effects are observed in the wing by overexpression of Vari in a WT background. Wing hairs retain a parallel alignment while pointing distally (H). In a null mutant background, imposed expression of Vari results in abnormal wing hair alignment (arrowhead, G). While some hairs point distally, patches of hairs point in random directions with irregular alignments when compared with neighbouring hairs. If Vari levels are reduced in developing wing tissue, as in *UAS-variIR; dpp-GAL4*, flies, patches of wing hairs with mis-aligned hairs, and un-extended hairs (arrowhead) are seen. Wings mosaic for *vari *also have patches of un-extended hairs (arrowhead, J). Overall morphology of all wings is similar to the WT (insets). (K) Mosaic patches on the dorsal thorax reveals patches of naked cuticle, and duplicated bristles (arrowhead). WT, wildtype. Magnification: A-C: 140×; A'-C, F': 300×; D, E: 450×; G-J: 20×; K: 50×.

We examined adult epithelial structures for developmental abnormalities and found disruptions in ommatidial patterning and wing hair alignment. Overexpression of Vari in a null mutant background results in missing ommatidia or extra interommatidial bristles (IOBs) (arrow, Fig. 7B'). These phenotypes are not observed if Vari is overexpressed in either a heterozygous mutant (Fig. 7A) or wildtype background (Fig. 7C), suggesting that the rescue phenotype results from incomplete restoration of Vari function in the rescue. The *daughterlessGAL4 *stock has a separate phenotype of missing (arrowhead, Fig. 7A'–C') or displaced IOBs (asterisk, Fig. 7A'–C') also revealed in controls. To explore the requirement of *vari *for ommatidial development, we generated patches of *vari*^48*EP *^null tissue using the FLP/FRT approach. We employed a GFP marker distal to the 40A FRT insert so that we might identify mutant clones by their lack of GFP expression. Recombination was induced in mitotic cells by heat shock induced Flippase expression, once, early in each larval instar. This treatment was lethal for 65% of the potential mosaics (n = 146), indicating that for many clones, *vari *was required for required for vital functions. All adult ommatidia expressed GFP in our experiments, suggesting that *vari *null clones did not succeed in contributing to adult ommatidia. All eyes had either small domains of fused ommatidia and/or duplicated bristles, not seen in controls (Fig 7D–F), suggesting that patterning of the remaining cells was affected.

SJ components Gliotactin (Gli) and Cora are required for proper hair alignment in the adult wing [50]. We examined overall wing morphology of adult flies overexpressing Vari in a null mutant background (inset, Fig. 7G) and found it to be similar to the imposed expression on a wildtype wing (inset, Fig. 7H). However, wing hair alignment is abnormal. Unlike the control wing (Fig. 7H), rescued adults show patches of wing hairs with abnormal alignment compared to their neighbouring hairs (arrowheads, Fig. 7G). The abnormal hair alignment was observed in unmounted wings, eliminating the possibility of a mounting artifact. Similar effects were seen in wings where Vari levels were reduced by expression of *vari-IR (UAS-vari-IR; dpp-GAL4*), and upon the generation of *vari *null patches in the wing with FLP/FRT. In both cases, domains of misaligned hairs are associated with patches of wing cells with incomplete hair extension (Fig. 7I, J).

We examined the polarity of bristles on the thorax, abdomen and legs of rescued adults (data not shown) and did not detect abnormal polarization, or multiple hairs per cell. From these results, we suspect that the misalignment phenotype is not due to a disruption in planar cell polarity, but rather due to altered cell polarization common to *vari*, *cora *and *gli *mutants [50]. We rarely observed mutant patches of body cuticle of FLP/FRT mosaics. These patches were associated with loss of hairs, multiple bristles from fused sockets, and small breaches of the cuticle (Fig. 7K).

## Discussion

Our study, and others [32,34] have characterised a MAGUK family member encoded by *Drosophila *CG9326, Varicose. We have shown that Varicose localizes to pSJs of all embryonic ectodermally-derived epithelial tissues as well as the pSJs of the PNS. We have detected Vari expression in the neuroepithelium of the developing optic lobe in a non-junction associated pattern, which is unique for a MAGUK member. Expression of Varicose in a subset of central brain neuroblasts and differentiated neurons of the adult nervous system emphasizes the importance and versatility of its function throughout development. Mutations in *vari *result in mislocalized SJ markers and disruption of the paracellular seal. Loss-of-function *vari *alleles display dilated and contorted tracheal tubes, implicating *vari *in tracheal morphogenesis. Furthermore, genetic mosaic and partial rescue phenotypes in the eye and wing suggests a role for *vari *during adult epithelial morphogenesis.

### Varicose plays a role in septate junction assembly

We have presented here several lines of evidence demonstrating that Varicose is required for septate junction formation. First, Varicose co-localizes with known SJ protein NrxIV in all embryonic pSJs, and NrxIV is mislocalized in the absence of *varicose *activity. Second, in *vari *null mutants, SJ do not mature to the point of septa formation. Third, the transepithelial barrier of *vari *mutants is 'leaky' to tracer dyes.

Embryos mutant for *varicose *show mislocalization of SJ proteins like NrxIV, FasIII and the Na^+^K^+^ATPase basally along the plasma membrane of epithelia (this report and Wu et al., [32]). The localization of SJ protein Dlg was not affected however, indicating Vari is not required to establish epithelial polarity. This is not a surprising result as the onset of Varicose expression appears midway through embryogenesis, a time when polarity has already been established and SJs begin to assemble [8]. Proper localization of Vari requires Nrg. Varicose has been shown to interact with NrxIV [32,34] and all three proteins share an mutually-dependent relationship necessary for proper subcellular localization [51]. Vari reduces the lateral mobility of Nrg and NrxIV [51], suggesting that in the absence of *vari*, the assembly of key SJ proteins is interrupted, disrupting the architecture of the junctional region and triggering a cascade of mislocalized proteins.

To date, all junctional proteins expressed in embryonic epithelia are also expressed in imaginal discs. While this work was in review, Bachmann and colleagues provided immunocytochemical evidence for Vari expression in the wing and eye discs [34].

Our ultrastructural analysis indicates that embryos null for *vari *die before intermembrane septa develop. Bachmann et al., [34] establish that septa do not develop in hypomorphs that develop further as embryos. *nrxIV *and *cora *mutants also lack septa, which are proposed to have a sealing function in the transepithelial barrier [15,18]. An affinity approach has identified NrxIV as a potential Vari binding partner [32]. These data are consistent with the failure of *vari *mutants to exclude dye in embryonic trachea. In contrast, SJ mutants, *gliotactin *and *sinuous *(*sinu*) show defects in septa array and septa number, respectively [52,53]. Mutations in *vari *enhance the *sinu *phenotype [52]. Together these results suggest Vari, like NrxIV and Cora, functions in assembling septa strands. However, the low levels of Vari in larvae suggest that Vari is not essential to maintain SJ.

SJ integrity in *Drosophila *requires Megatrachea (Mega), a claudin that has a C-terminal PDZ binding domain [54]. It has been suggested that a MAGUK member may act to tether Mega to the NrxIV/Cora complex to assemble the SJ [19,54]. We propose Vari as a candidate for this function.

### A role for MAGUKs in the nervous system?

Expression of Vari in peripheral glia, but not the perineural sheath or midline glia of the embryonic nervous system is consistent with function in the establishment of ectodermally-derived pSJs. Neural expression was not detected in embryos. However, the distribution of Vari in the late larval and adult central nervous system suggests non-junctional roles for this MAGUK. In the optic lobe NE, which does express Dlg, Vari expression overlaps, and extends into the apical cytoplasm. Vari is not expressed in NBs of the embryo and medial optic lobe, yet is expressed in the cytoplasm of some central NBs of late third instar, and in low levels in the soma of differentiated neurons. This pattern of expression is not typical of other junctional or cell polarity markers like Bazooka, Glaikit or Miranda [55-57], or of MAGUKs in general and must be clarified by further study. This issue may be approached with nervous system specific RNAi knockdown of Vari, which we found to be pupal lethal (data not shown).

Several independent lines of evidence suggest that our serum is specific to Varicose. First, our epithelial expression pattern observed during embyrogenesis is consistent with previous reports on *varicose *[32]. Second, a null allele, *vari*^48*EP*^, lacks wildtype Varicose imunolabeling. Third, the NE expression pattern is absent when third instar larval brains are labeled with pre-immune sera. Fourth, our antibody detects Varicose expression in the ventral midline when *UAS-vari *is mis-expressed in the midline using *single-minded GAL4*. Fifth, although normal protein levels are at the threshold of detection, over-expression of *UAS-var*i using heat-shock *GAL4 *provides ample protein to be detected by western blotting (see Additional file 2).

### Varicose is involved in regulating tube size

The *Drosophila *tracheal system is a well developed model for the dissection of pathways regulating tube formation [31]. pSJ components are implicated in the regulation of tubule size. Genes regulating tube size fall into two phenotypic categories; those required to regulate tube length and those required for normal tube diameter (reviewed in [58]). Several lines of evidence have suggested that pSJ components are involved in regulating tube length. Mutations in genes for SJ proteins like *mega*, *sinu *and the Na^+^K^+^ATPase β subunit, *nrv2 *have tortuous and elongated tracheal trunks, without affecting tube diameter [52,54,59]. In contrast, mutations in *vari *do not appear to affect tube length. Tracheal tubes in *vari *mutants have irregular and enlarged tube diameters reminiscent of mutations affecting *mmy/cystic *and *kkv*, enzymes required for chitin synthesis [60,61]. Epistatic analysis of *vari *and *sinu *reveals a tracheal phenotype in double mutants that is worse than either single mutant, suggesting these proteins function in different pathways [52].

The chitin matrix is secreted from the apical surface of tracheal cells and synthesis of the matrix has been linked to controlling tube diameter. During expansion of the dorsal trunk, the cylinder expands as the lumen dilates [46]. It is suggested that formation of the chitin matrix is needed for the organized radial expansion of tracheal tubes [61]. Tracheal enlargement in *vari *is similar to *cystic *and *kkv *mutants, and all three have reduced deposition of 2A12 antigen [61]. Wu and colleagues [32] further show that *vari *mutants fail to secrete apical protein Serpentine and secrete variable amounts of Vermiform. Unexpectedly, cuticle ultrastructure and taenidial ridges appear normal in *vari *mutants. This result is unlike tracheal mutants affecting tube length, such as *sinu*, where taenidial folds are irregular [52]. Our data suggests that lumenal protein secretion in *vari *mutants is sufficient to produce cuticle and regulate tube length, and that the SJ may also play a role in regulating tube diameter.

### MAGUKs are involved in morphogenesis of adult tissues

Our knowledge of the function of septate junctions during metamorphosis is limited, however a role for SJs in the adult ommatidium has been described. The SJ component NrxIV was shown to localize to junctional regions in the pupal and adult eye. Loss of *nrxIV *disrupts SJ function, which leads to structural disorganization resulting from a loss of adhesion between cells of the adult ommatidia [13]. Although *nrxIV*^- ^clones survive in the adult eye [13], *vari*^- ^clones do not, indicating that *vari *has functions other than localizing NrxIV. Partial restoration of *vari *(by transgene rescue) during adult morphogenesis results in missing ommatidia and irregular bristle patterning. Vari may spatially organise adhesions during this developmental process, and reduced levels of Vari disrupts epithelial patterning. Vari levels are much lower subsequent to *vari-RNAi *treatment described by Bachmann et al., [34]. The retinal and wing epithelia survive, but epithelial patterning is more disorganised. We report that mosaic null clones of *vari *generate duplicated bristles in the eye, and clumped sensory hairs on the thorax. The failure to establish SJs in these clones may result in delaminating mutant cells adopting a neurogenic fate, and thereby generate extra sensory structures.

The involvement of SJs during wing imaginal disc to adult wing morphogenesis is also unclear. However, two known SJ components, Gli and Cora are required for survival of pre-hair cells during pupation [18,50]. Similar to mutations in *gli *and *cora*, our partial rescue of *vari*, and RNAi of *vari *resulted in patches of wing hairs that fail to point distally [50]. Although reminiscent of a Frizzled (Fz) planar cell polarity phenotype, the mechanism regulating hair alignment acts independently of Fz. Patches of wing hairs, although not pointing distally, retain a parallel alignment with neighbouring hairs in *Fz *mutants [62,63]. This is unlike the random alignment seen in *vari*, *gli*, and *cora *mutants where polarity of neighbouring wing hairs is different. During pupal development, the position and orientation of wing prehairs are determined and then stabilized during later stages [50]. Vari and other SJ proteins play a role in prehair patterning.

## Conclusion

The MAGUK protein PALS2 has been proposed to act in scaffold formation at the basolateral membrane of mammalian epithelia [30]. Here we show that a *Drosophila *homologue, Vari, is similarly distributed, and is required in ectodermally-derived epithelia to elaborate pSJs and establish a paracellular barrier. Embryos lacking *vari *function display mislocalization of essential pSJ membrane proteins, including NrxIV, Na^+^K^+^ATPase and FasIII, and are unable to control the permeability of the tracheal membrane. As a result, the trachea fail to fill with air, and the embryos die in early stage 17. The function of SJs in the morphogenesis of the wing and eye is less well characterised, yet imaginal epithelia lacking *vari*, *cora *or *gli *do not survive to the adult. The eye and wing phenotypes of reduced *vari *function overlaps with patterning defects of mutations in SJ genes *nrxIV*, *gli *and *cora*. Together, they indicate an uncharacterised role for SJs in establishing pattern in epithelial sheets. Vari is not expressed in the embryonic central nervous system, but is expressed apically in the neuroepithelium of the optic lobes and in neuronal cell bodies. These structures do not have pSJs, and indicate that there are uncharacterised functions of Vari, distinct from a role in the assembly of cell junctions.

## Methods

### *Drosophila *stocks

Canton-S P was used as a wildtype control. EP-element insertion line GE13049 was obtained from GenExel, Inc. Stocks were backcrossed to ensure clean backgrounds. *vari*^48*EP*^, *vari*^*B*4^, *vari*^*K*4^, *vari*^*L*4 ^and *vari*^*B*5 ^generated by imprecise excision of GE13049. *vari*^48*EP *^is a protein null allele (this study). *Df(2L)Exel*^7079 ^deficiency deletes 19 genes in addition to *vari*. *Single-minded *GAL4 was obtained from John Nambu. *UAS-vari-IR *(transformant 24156) was obtained from the Vienna *Drosophila *RNAi Centre[48]. All other stocks were obtained from the Bloomington *Drosophila *Stock Centre.

### Antibody production

Rat polyclonal antibody was produced against Vari. Total RNA was extracted from Canton-S P adults using TRIzol (Invitrogen). cDNA synthesis was performed using Ready-To-Go RT-PCR Beads (Amersham). A 909 bp fragment was amplified using the sense primer 5'-GCAAGATCTAGTGGACGACGAATAATCAAG-3' and the antisense primer 5'-GATGGATTCCGGTTGGAGCCCGTGG-3'. This cDNA fragment, corresponding to amino acid residues 83–386, was fused to a C-terminal his-tag in pET29b(+) (Novagen) and expressed in BL21DE3. Following induction, the fusion protein was purified under denaturing conditions by affinity chromatography using His-Select Nickel Affinity Gel (Sigma) and used for rat immunization. Polyclonal antiserum was affinity purified using CNBr-activated sepharose according to manufacturer's protocol (Amersham).

### Immunohistochemistry

Immunohistochemistry techniques were adapted from Patel [64]. Embryos were collected, decorionated, fixed and incubated in primary antibody diluted in phosphate-buffered saline (PBS) containing 0.5% Triton X-100 and 10% normal goat serum (NGS). Primary antibodies were used at the following dilutions: rat anti-Vari (1:15) (this study), rabbit anti-NrxIV (1:300) (gift from H.J. Bellen, Baylor College of Medicine, Houston, TX), chicken anti-βgalactosidase (1:150) [65], and anti-Phosphotyrosine (1:300) (Millipore). The following monoclonal antibodies were obtained from the Developmental Studies Hybridoma Bank: anti-Crumbs (1:30), anti-αSpectrin (1:30), anti-Discs Large (1:30), anti-Na^+^K^+^ATPase (1:300), MAb2A12 (1:30), anti-Repo (1:7), anti-Elav (1:75), anti-Prospero (1:4), and anti-FasIII (1:30). Embryos were incubated in fluorescent secondary (1:150 dilution, Alexa 488, Alex 594; Molecular Probes). Anti-βgal was detected using biotinylated secondary antibody (1:150) (Vector Laboratories) followed by incubation with Vector Laboratories Elite ABC and 3, 3-Diaminobenzidine Tetra hydrochloride (DAB, Gibco-BRL). Embryos were visualized by confocal microscopy using Zeiss LSM510 or Zeiss Axioskop microscope. Images were processed using ImageJ and Adobe Photoshop.

### Tissue dissection

Third instar larvae brains and pupae brains (50 hours after inclusion) were dissected in PBS and fixed in 4% paraformaldehyde. Following several washes in PBS with 0.3% Triton X-100, tissues were incubated in primary antibody as stated above. Anti-FasII was diluted 1:30 in PBS with 0.5% Triton X-100 and 10% NGS.

Wings were prepared as described in Settle, et al. [66].

### Transgenic constructs

Total RNA was extracted from Canton-S P adults using TRIzol (Invitrogen). Reverse transcription was performed using M-MLV Reverse Transcriptase and random decamers (Ambion). Full-length Vari was amplified using the sense primer 5'-CCGAGGACGTCCTCTAGACCAAGATGCCAG-3' and the antisense primer 5'-CCCCGGAGGGCGCATCTAGACTTATACAAACATTGC-3' to amplify a cDNA fragment corresponding to amino acid residues 1–469. This cDNA was cloned into pUASt and injected into embryos using standard techniques.

### Mosaics

Mosaic clones of *vari *mutant cells were generated using a FLP/FRT-mediated technique [67]. Mitotic recombination of our null allele, *vari*^48*EP*^, was induced by treating *yw-, hsFLP; vari*^48*EP*^, *FRT40A/ubi-GFP, FRT40A *larvae 24, 48 and 96 hours after egg laying to a single heat shock at 37°C for 1 hour. Flies were raised at 25°C prior to and following heat shock treatment. Mutant clones in the adult eye and thorax were visualized by scanning electron microscopy as described below. To visualize wing defects, whole flies were dehydrated using an ethanol gradient and stored in methyl salicylate. Wings were mounted in D.P.X. on microscope slides and sealed with coverslips. Photomicrographs were processed using OpenLab and Adobe Photoshop^® ^7.0.

### Electron microscopy

Stage 17 embryos were injected with 5% glutaraldehyde in 0.05 M Cacodylate buffer (pH 7.2) as described [68], post-fixed in 1.0% Osmium tetroxide in dH_2_O and stained *en bloc *with aqueous 2% uranyl acetate. Embryos were dehydrated, embedded and sectioned with established methods [69]. Four embryos of each genotype were sectioned for analysis.

### Scanning electron microscopy

Imaging of compound eyes and thorax was performed as described in Settle et al., [66]. In brief, cold anaesthetised adults were imaged at 3.0 Torr in an Electroscan 2020 Environmental Scanning Electron Microscope.

### Dye permeability assay

Fluorescent dye injection was performed as described [18]. Stage 17 embryos were examined within 30 minutes of injection on a Zeiss LSM510. Mutants were identified by lack of GFP expression from the balancer (*CyO, Kr-GAL4, UAS-GFP*).

## Abbreviations

CNS: central nervous system; GFP green fluorescent protein; IOB: interommaditial bristles; MAGUK: membrane associated guanylate kinase; FLP/FRT: Flippase/Flippase recombination target; NE: neuro-epithelium; PALS: Proteins Associated with Lin-7; PDZ: post synaptic density protein, disc large tumor suppressor, zonula occludens-1 protein; PNS: peripheral nervous system; PSJ: pleated septate junction; SH3: src homology 3; SJ: septate junction; TJ: tight junction; UTR: untranslated region; WT: wildtype.

## Authors' contributions

KEM carried out all of the genetic and phenotype studies, except the dye injection and electron microscopy, performed by JRJ. KEM drafted the manuscript, revised by JRJ.

## Supplementary Material

Additional file 1 **Varicose localizes to the septate junction of embryonic ectodermally-derived epithelia.** Whole-mount WT embryos labeled with Vari and visualized by confocal microscopy. (A) Vari is detected in epithelial cells of the trachea (arrowhead), hindgut (arrow) and epidermis (*). (B-G) The hindgut of WT embryos, labeled with Vari (green) and lateral membrane markers (red). (B, C) Vari localizes basal to the subapical region, shown by the lack of overlap with Phosphotyrosine (B) and Crumbs (C). (D) Vari overlaps at the apical membrane with alpha-Spectrin (yellow) but is excluded from the basal membrane. Vari (E) is restricted to SJs, shown by co-localization with SJ markers Dlg (F, merge G; yellow). All embryos are stage 15. WT, wildtype. Calibration: 50 μm, A; 2 μm, B-G. Click here for file

Additional file 2 **Expression patterns observed are specific to Varicose.** To ensure specificity of our Vari antibody, whole-mount *vari*^48*EP *^embryos and visualized by confocal microscopy. Homozygous embryos were selected by the absence of balancer GFP expression. (A) The WT Varicose expression pattern seen in the balancer controls (asterisk) were not observed in embryos homozygous for null allele *vari*^48*EP *^(arrow). Vari expression is not detected in neuroepithelial cells immunolabeled with pre-immune sera (B) and Dlg (C; merge, D). To further establish antibody specificity, we mis-expressed *UAS*-*vari *in the mesectoderm, using *single-minded GAL4*. When mis-expressed, Varicose was seen in the embryonic midline of embryos labeled with anti-Vari (arrowhead, F) whereas midline expression was absent in WT embyos (E). Ventral view, anterior to the left. (G) We over-expressed UAS-*vari *using heat-shock *GAL4 *to visualize Varicose protein levels by Western blotting. In contrast to low protein levels in *HS-GAL4 *or *UAS-Vari *parentalcontrols, over-expression (in *HS-GAL4; UAS-Vari *embryos) substantially elevates detected Vari protein. WT, wildtype. Calibration: A-50 μm; B-D 10 μm. Click here for file

Additional file 3 **Varicose expression in imaginal discs.** Third instar larval discs were dissected and double immunolabeled with Vari post-immune serum (green) and Dlg (red) (A-L) or pre-immune serum (green) and Dlg (red) (A'-L'). Gain levels for images A'-L' were increased in order to visualize possible immunolabeling. We did not detect differences in the pattern of labeling between and pre and post-immune sera in antennal (A-C), eye (D-F), leg (G-I) or wing (J-L) discs. Varicose expression may be low in the eye discs (D-F), and the characteristic SJ labeling pattern was not observed. Images are a single section, visualized by confocal microscopy. Calibration: A-F, 5 μm; G-L, 2 μm. Click here for file

Additional file 4 **Tracheal development requires Varicose.** (A-H) The tracheal lumen of early stage 16 *vari *mutant embryos were labeled with MAb2A12. In wildtype (A) embryos, the diameter of the Dorsal Trunk (DT) is uniform, and the Lateral Trunk (LT) is continuous with the DT. All *vari *mutants (B-G, and heteroallelic H) exhibit large dilations along the DT and LT. Lumenal staining is reduced in all *vari *alleles in comparison to wildtype and control. Lateral view: anterior to the left, dorsal is up. Calibration: 20 μm, A, B; and 20 μm C-H. Click here for file

## References

[B1] Erez N, Bershadsky A, Geiger B (2005). Signaling from adherens-type junctions. Eur J Cell Biol.

[B2] Knust E, Bossinger O (2002). Composition and formation of intercellular junctions in epithelial cells. Science.

[B3] Tsukita S, Furuse M, Itoh M (2001). Multifunctional strands in tight junctions. Nat Rev Mol Cell Biol.

[B4] Matter K, Mellman I (1994). Mechanisms of cell polarity: sorting and transport in epithelial cells. Curr Opin Cell Biol.

[B5] D'Souza-Schorey C (2005). Disassembling adherens junctions: breaking up is hard to do. Trends Cell Biol.

[B6] Matter K, Balda MS (2003). Functional analysis of tight junctions. Methods.

[B7] Tepass U, Hartenstein V (1994). The development of cellular junctions in the Drosophila embryo. Dev Biol.

[B8] Tepass U, Tanentzapf G, Ward R, Fehon R (2001). Epithelial cell polarity and cell junctions in Drosophila. Annu Rev Genet.

[B9] Carlson SD, Juang JL, Hilgers SL, Garment MB (2000). Blood barriers of the insect. Annu Rev Entomol.

[B10] Staehelin LA (1973). Further observations on the fine structure of freeze-cleaved tight junctions. J Cell Sci.

[B11] Hortsch M, Margolis B (2003). Septate and paranodal junctions: kissing cousins. Trends Cell Biol.

[B12] Parker RJ, Auld VJ (2006). Roles of glia in the Drosophila nervous system. Semin Cell Dev Biol.

[B13] Banerjee S, Bainton RJ, Mayer N, Beckstead R, Bhat MA (2008). Septate junctions are required for ommatidial integrity and blood-eye barrier function in Drosophila. Dev Biol.

[B14] Woods DF, Bryant PJ (1991). The discs-large tumor suppressor gene of Drosophila encodes a guanylate kinase homolog localized at septate junctions. Cell.

[B15] Baumgartner S, Littleton JT, Broadie K, Bhat MA, Harbecke R, Lengyel JA, Chiquet-Ehrismann R, Prokop A, Bellen HJ (1996). A Drosophila neurexin is required for septate junction and blood-nerve barrier formation and function. Cell.

[B16] Fehon RG, Dawson IA, Artavanis-Tsakonas S (1994). A Drosophila homologue of membrane-skeleton protein 4.1 is associated with septate junctions and is encoded by the coracle gene. Development.

[B17] Genova JL, Fehon RG (2003). Neuroglian, Gliotactin, and the Na+/K+ ATPase are essential for septate junction function in Drosophila. J Cell Biol.

[B18] Lamb RS, Ward RE, Schweizer L, Fehon RG (1998). Drosophila coracle, a member of the protein 4.1 superfamily, has essential structural functions in the septate junctions and developmental functions in embryonic and adult epithelial cells. Mol Biol Cell.

[B19] Ward REt, Lamb RS, Fehon RG (1998). A conserved functional domain of Drosophila coracle is required for localization at the septate junction and has membrane-organizing activity. J Cell Biol.

[B20] Bieber AJ, Snow PM, Hortsch M, Patel NH, Jacobs JR, Traquina ZR, Schilling J, Goodman CS (1989). Drosophila neuroglian: a member of the immunoglobulin superfamily with extensive homology to the vertebrate neural adhesion molecule L1. Cell.

[B21] Dubreuil RR, Maddux PB, Grushko TA, MacVicar GR (1997). Segregation of two spectrin isoforms: polarized membrane-binding sites direct polarized membrane skeleton assembly. Mol Biol Cell.

[B22] Funke L, Dakoji S, Bredt DS (2005). Membrane-associated guanylate kinases regulate adhesion and plasticity at cell junctions. Annu Rev Biochem.

[B23] Dimitratos SD, Woods DF, Stathakis DG, Bryant PJ (1999). Signaling pathways are focused at specialized regions of the plasma membrane by scaffolding proteins of the MAGUK family. Bioessays.

[B24] Kaech SM, Whitfield CW, Kim SK (1998). The LIN-2/LIN-7/LIN-10 complex mediates basolateral membrane localization of the C. elegans EGF receptor LET-23 in vulval epithelial cells. Cell.

[B25] Caruana G (2002). Genetic studies define MAGUK proteins as regulators of epithelial cell polarity. Int J Dev Biol.

[B26] Gonzalez-Mariscal L, Betanzos A, Avila-Flores A (2000). MAGUK proteins: structure and role in the tight junction. Semin Cell Dev Biol.

[B27] Kamberov E, Makarova O, Roh M, Liu A, Karnak D, Straight S, Margolis B (2000). Molecular cloning and characterization of Pals, proteins associated with mLin-7. J Biol Chem.

[B28] Bachmann A, Schneider M, Theilenberg E, Grawe F, Knust E (2001). Drosophila Stardust is a partner of Crumbs in the control of epithelial cell polarity. Nature.

[B29] Wang Q, Hurd TW, Margolis B (2004). Tight junction protein Par6 interacts with an evolutionarily conserved region in the amino terminus of PALS1/stardust. J Biol Chem.

[B30] Shingai T, Ikeda W, Kakunaga S, Morimoto K, Takekuni K, Itoh S, Satoh K, Takeuchi M, Imai T, Monden M, Takai Y (2003). Implications of nectin-like molecule-2/IGSF4/RA175/SgIGSF/TSLC1/SynCAM1 in cell-cell adhesion and transmembrane protein localization in epithelial cells. J Biol Chem.

[B31] Beitel GJ, Krasnow MA (2000). Genetic control of epithelial tube size in the Drosophila tracheal system. Development.

[B32] Wu VM, Yu MH, Paik R, Banerjee S, Liang Z, Paul SM, Bhat MA, Beitel GJ (2007). Drosophila Varicose, a member of a new subgroup of basolateral MAGUKs, is required for septate junctions and tracheal morphogenesis. Development.

[B33] Moyer K, Jacobs J (2007). Senz'aria, a MAGUK family adapter, is required for tracheal morphogenesis. A Dros Res Conf.

[B34] Bachmann A, Draga M, Grawe F, Knust E (2008). On the role of the MAGUK proteins encoded by Drosophila varicose during embryonic and postembryonic development. BMC Dev Biol.

[B35] Crosby MA, Goodman JL, Strelets VB, Zhang P, Gelbart WM (2007). FlyBase: genomes by the dozen. Nucleic Acids Res.

[B36] Lee S, Fan S, Makarova O, Straight S, Margolis B (2002). A novel and conserved protein-protein interaction domain of mammalian Lin-2/CASK binds and recruits SAP97 to the lateral surface of epithelia. Mol Cell Biol.

[B37] Tepass U (1996). Crumbs, a component of the apical membrane, is required for zonula adherens formation in primary epithelia of Drosophila. Dev Biol.

[B38] Woods DF, Wu JW, Bryant PJ (1997). Localization of proteins to the apico-lateral junctions of Drosophila epithelia. Dev Genet.

[B39] Xiong WC, Okano H, Patel NH, Blendy JA, Montell C (1994). repo encodes a glial-specific homeo domain protein required in the Drosophila nervous system. Genes Dev.

[B40] Egger B, Boone JQ, Stevens NR, Brand AH, Doe CQ (2007). Regulation of spindle orientation and neural stem cell fate in the Drosophila optic lobe. Neural Develop.

[B41] Doe CQ, Chu-LaGraff Q, Wright DM, Scott MP (1991). The prospero gene specifies cell fates in the Drosophila central nervous system. Cell.

[B42] Peng CY, Manning L, Albertson R, Doe CQ (2000). The tumour-suppressor genes lgl and dlg regulate basal protein targeting in Drosophila neuroblasts. Nature.

[B43] Robinow S, Campos AR, Yao KM, White K (1988). The elav gene product of Drosophila, required in neurons, has three RNP consensus motifs. Science.

[B44] Lundgren J, Masson P, Mirzaei Z, Young P (2005). Identification and characterization of a Drosophila proteasome regulatory network. Mol Cell Biol.

[B45] Fristrom DK (1982). Septate junctions in imaginal disks of Drosophila: a model for the redistribution of septa during cell rearrangement. J Cell Biol.

[B46] Luschnig S, Batz T, Armbruster K, Krasnow MA (2006). serpentine and vermiform encode matrix proteins with chitin binding and deacetylation domains that limit tracheal tube length in Drosophila. Curr Biol.

[B47] Wang S, Jayaram SA, Hemphala J, Senti KA, Tsarouhas V, Jin H, Samakovlis C (2006). Septate-junction-dependent luminal deposition of chitin deacetylases restricts tube elongation in the Drosophila trachea. Curr Biol.

[B48] Dietzl G, Chen D, Schnorrer F, Su KC, Barinova Y, Fellner M, Gasser B, Kinsey K, Oppel S, Scheiblauer S (2007). A genome-wide transgenic RNAi library for conditional gene inactivation in Drosophila. Nature.

[B49] Xu T, Rubin GM (1993). Analysis of genetic mosaics in developing and adult Drosophila tissues. Development.

[B50] Venema DR, Zeev-Ben-Mordehai T, Auld VJ (2004). Transient apical polarization of Gliotactin and Coracle is required for parallel alignment of wing hairs in Drosophila. Dev Biol.

[B51] Laval M, Bel C, Faivre-Sarrailh C (2008). The lateral mobility of cell adhesion molecules is highly restricted at septate junctions in Drosophila. BMC Cell Biol.

[B52] Wu VM, Schulte J, Hirschi A, Tepass U, Beitel GJ (2004). Sinuous is a Drosophila claudin required for septate junction organization and epithelial tube size control. J Cell Biol.

[B53] Schulte J, Tepass U, Auld VJ (2003). Gliotactin, a novel marker of tricellular junctions, is necessary for septate junction development in Drosophila. J Cell Biol.

[B54] Behr M, Riedel D, Schuh R (2003). The claudin-like megatrachea is essential in septate junctions for the epithelial barrier function in Drosophila. Dev Cell.

[B55] Dunlop J, Morin X, Corominas M, Serras F, Tear G (2004). glaikit is essential for the formation of epithelial polarity and neuronal development. Curr Biol.

[B56] Kuchinke U, Grawe F, Knust E (1998). Control of spindle orientation in Drosophila by the Par-3-related PDZ-domain protein Bazooka. Curr Biol.

[B57] Shen CP, Jan LY, Jan YN (1997). Miranda is required for the asymmetric localization of Prospero during mitosis in Drosophila. Cell.

[B58] Kerman BE, Cheshire AM, Andrew DJ (2006). From fate to function: the Drosophila trachea and salivary gland as models for tubulogenesis. Differentiation.

[B59] Paul SM, Ternet M, Salvaterra PM, Beitel GJ (2003). The Na+/K+ ATPase is required for septate junction function and epithelial tube-size control in the Drosophila tracheal system. Development.

[B60] Moussian B, Schwarz H, Bartoszewski S, Nusslein-Volhard C (2005). Involvement of chitin in exoskeleton morphogenesis in Drosophila melanogaster. J Morphol.

[B61] Araujo SJ, Aslam H, Tear G, Casanova J (2005). mummy/cystic encodes an enzyme required for chitin and glycan synthesis, involved in trachea, embryonic cuticle and CNS development – analysis of its role in Drosophila tracheal morphogenesis. Dev Biol.

[B62] Adler PN (2002). Planar signaling and morphogenesis in Drosophila. Dev Cell.

[B63] Wong LL, Adler PN (1993). Tissue polarity genes of Drosophila regulate the subcellular location for prehair initiation in pupal wing cells. J Cell Biol.

[B64] Patel NH (1994). Imaging neuronal subsets and other cell types in whole-mount Drosophila embryos and larvae using antibody probes. Methods Cell Biol.

[B65] MacMullin A, Jacobs JR (2006). Slit coordinates cardiac morphogenesis in Drosophila. Dev Biol.

[B66] Settle M, Gordon MD, Nadella M, Dankort D, Muller W, Jacobs JR (2003). Genetic identification of effectors downstream of Neu (ErbB-2) autophosphorylation sites in a Drosophila model. Oncogene.

[B67] Theodosiou NA, Xu T (1998). Use of FLP/FRT system to study Drosophila development. Methods.

[B68] Prokop A, Martin-Bermudo MD, Bate M, Brown NH (1998). Absence of PS integrins or laminin A affects extracellular adhesion, but not intracellular assembly, of hemiadherens and neuromuscular junctions in Drosophila embryos. Dev Biol.

[B69] Jacobs JR, Goodman CS (1989). Embryonic development of axon pathways in the Drosophila CNS. I. A glial scaffold appears before the first growth cones. J Neurosci.

