# Supplementary figures and images for: Varicose: a MAGUK required for the maturation and function of Drosophila septate junctions
Source: BMC Dev Biol. 2008 Oct 10;8:99. doi: 10.1186/1471-213X-8-99 (PMC2575209; doi:10.1186/1471-213X-8-99)

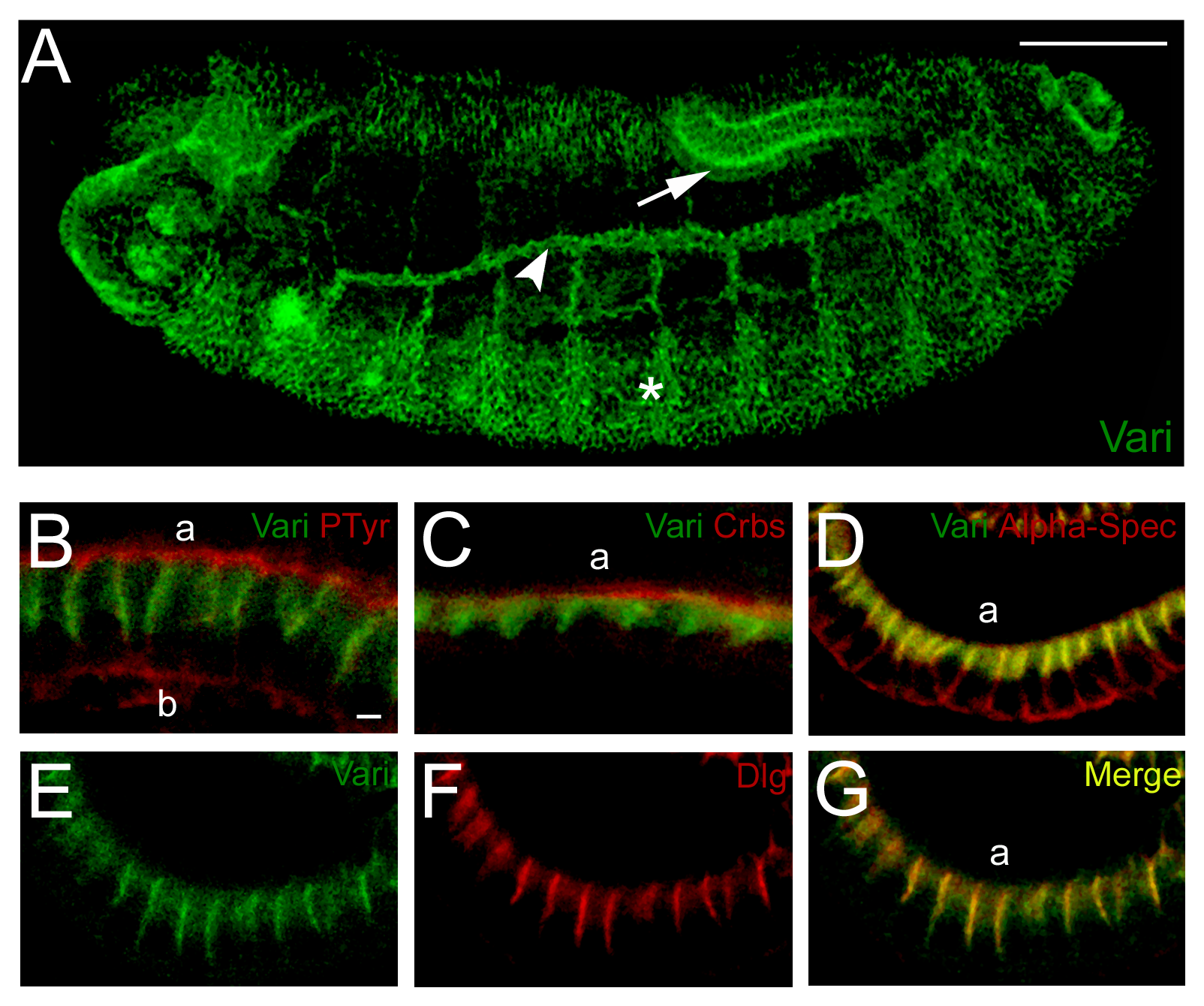

Supplement: Additional file 1 — Varicose localizes to the septate junction of embryonic ectodermally-derived epithelia. Whole-mount WT embryos labeled with Vari and visualized by confocal microscopy. (A) Vari is detected in epithelial cells of the trachea (arrowhead), hindgut (arrow) and epidermis (*). (B-G) The hindgut of WT embryos, labeled with Vari (green) and lateral membrane markers (red). (B, C) Vari localizes basal to the subapical region, shown by the lack of overlap with Phosphotyrosine (B) and Crumbs (C). (D) Vari overlaps at the apical membrane with alpha-Spectrin (yellow) but is excluded from the basal membrane. Vari (E) is restricted to SJs, shown by co-localization with SJ markers Dlg (F, merge G; yellow). All embryos are stage 15. WT, wildtype. Calibration: 50 μm, A; 2 μm, B-G. [file 1471-213X-8-99-S1.tiff]

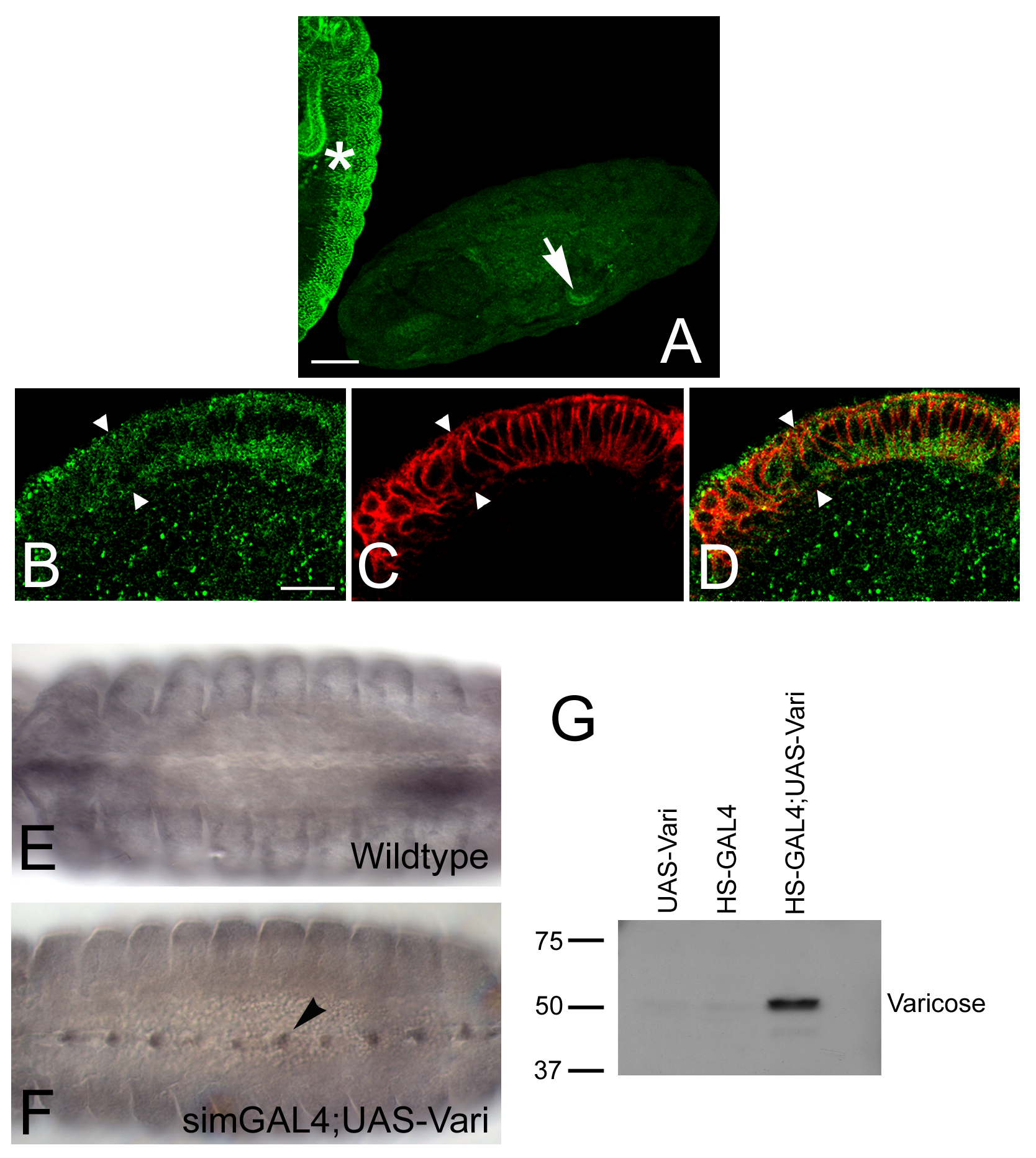

Supplement: Additional file 2 — Expression patterns observed are specific to Varicose. To ensure specificity of our Vari antibody, whole-mount vari48EP embryos and visualized by confocal microscopy. Homozygous embryos were selected by the absence of balancer GFP expression. (A) The WT Varicose expression pattern seen in the balancer controls (asterisk) were not observed in embryos homozygous for null allele vari48EP (arrow). Vari expression is not detected in neuroepithelial cells immunolabeled with pre-immune sera (B) and Dlg (C; merge, D). To further establish antibody specificity, we mis-expressed UAS-vari in the mesectoderm, using single-minded GAL4. When mis-expressed, Varicose was seen in the embryonic midline of embryos labeled with anti-Vari (arrowhead, F) whereas midline expression was absent in WT embyos (E). Ventral view, anterior to the left. (G) We over-expressed UAS-vari using heat-shock GAL4 to visualize Varicose protein levels by Western blotting. In contrast to low protein levels in HS-GAL4 or UAS-Vari parentalcontrols, over-expression (in HS-GAL4; UAS-Vari embryos) substantially elevates detected Vari protein. WT, wildtype. Calibration: A-50 μm; B-D 10 μm. [file 1471-213X-8-99-S2.tiff]

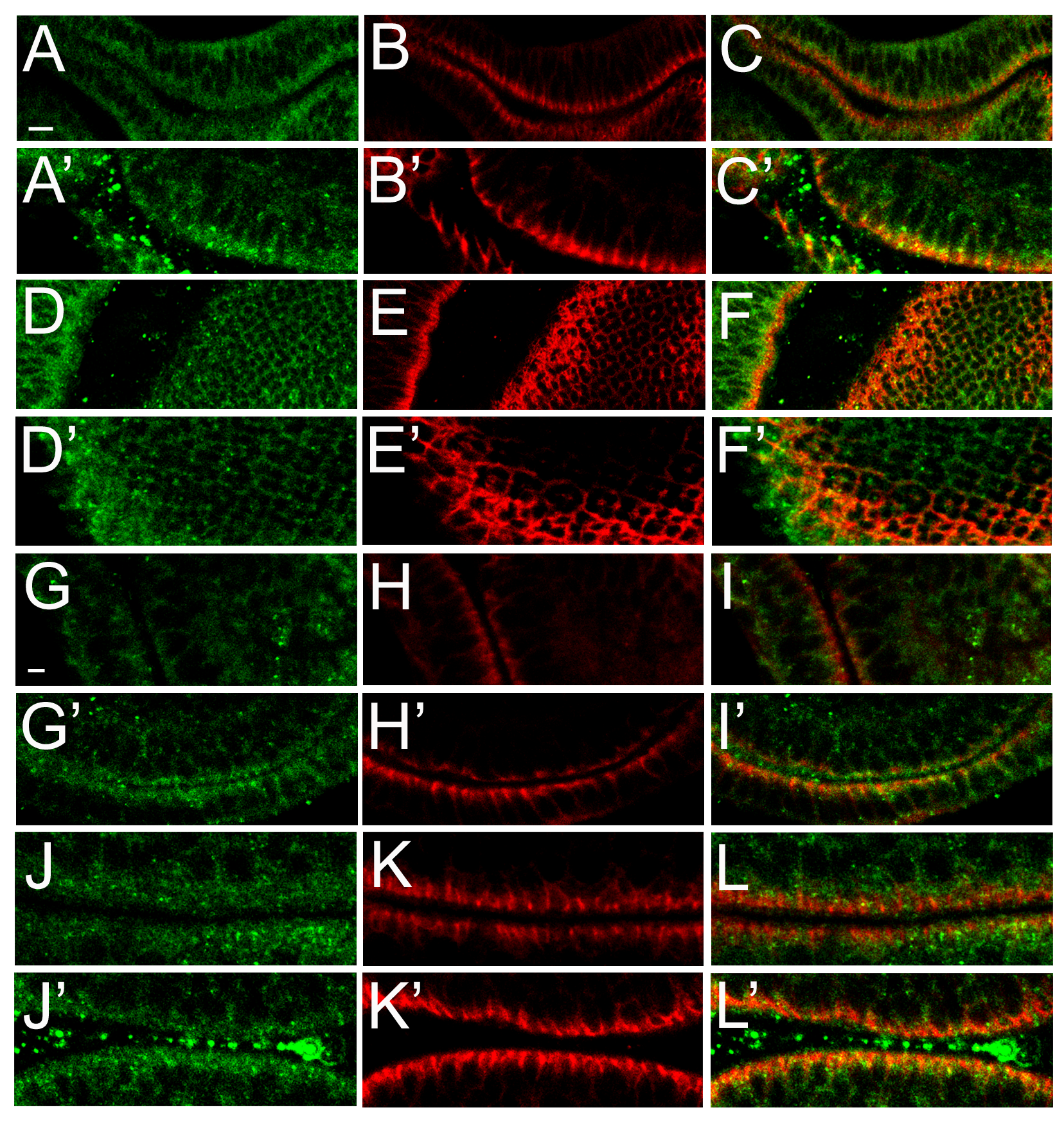

Supplement: Additional file 3 — Varicose expression in imaginal discs. Third instar larval discs were dissected and double immunolabeled with Vari post-immune serum (green) and Dlg (red) (A-L) or pre-immune serum (green) and Dlg (red) (A'-L'). Gain levels for images A'-L' were increased in order to visualize possible immunolabeling. We did not detect differences in the pattern of labeling between and pre and post-immune sera in antennal (A-C), eye (D-F), leg (G-I) or wing (J-L) discs. Varicose expression may be low in the eye discs (D-F), and the characteristic SJ labeling pattern was not observed. Images are a single section, visualized by confocal microscopy. Calibration: A-F, 5 μm; G-L, 2 μm. [file 1471-213X-8-99-S3.tiff]

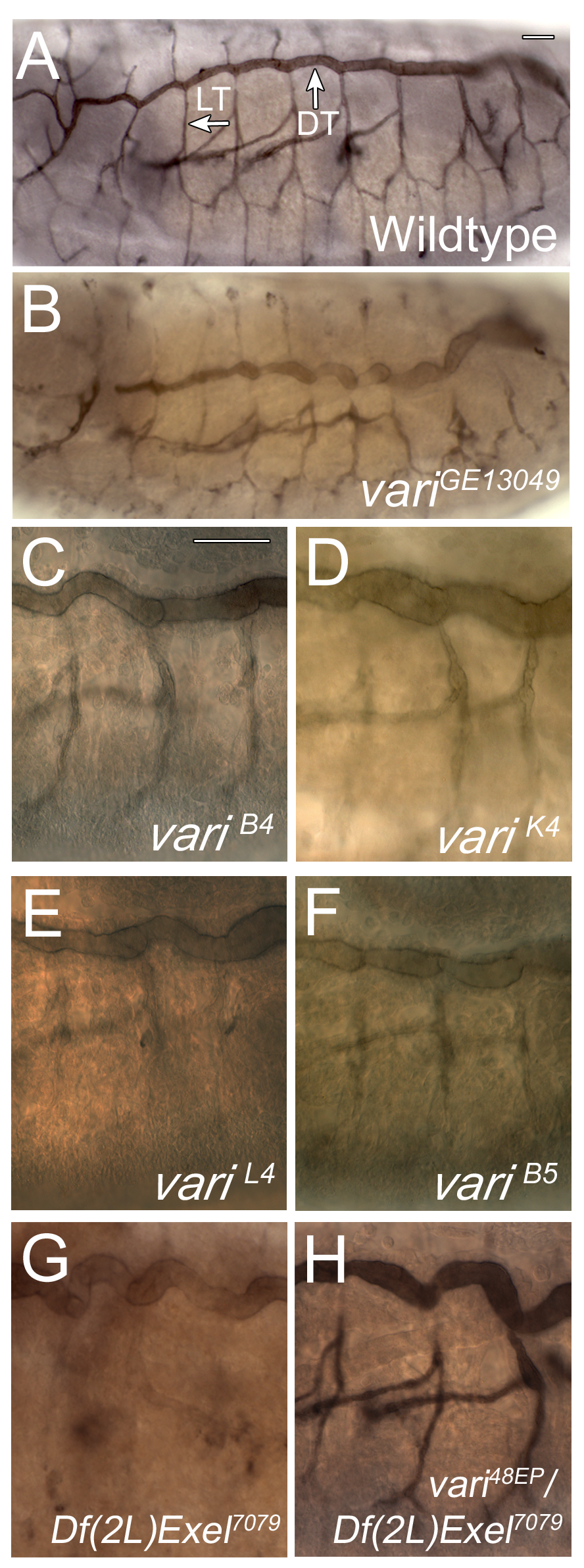

Supplement: Additional file 4 — Tracheal development requires Varicose. (A-H) The tracheal lumen of early stage 16 vari mutant embryos were labeled with MAb2A12. In wildtype (A) embryos, the diameter of the Dorsal Trunk (DT) is uniform, and the Lateral Trunk (LT) is continuous with the DT. All vari mutants (B-G, and heteroallelic H) exhibit large dilations along the DT and LT. Lumenal staining is reduced in all vari alleles in comparison to wildtype and control. Lateral view: anterior to the left, dorsal is up. Calibration: 20 μm, A, B; and 20 μm C-H. [file 1471-213X-8-99-S4.tiff]
